# In Situ Analytical Chemistry Laboratory for the Exobiology Extant Life Surveyor

**DOI:** 10.1021/acsmeasuresciau.5c00173

**Published:** 2026-03-12

**Authors:** Tomas Drevinskas, Morgan L. Cable, Christian Stenner, Alex S. Gardner, Michael Paton, Michael J. Malaska, Sarah Cruz, Richard Rieber, Rachel Etheredge, Matthew Robinson, Masahiro Ono, Maria F. Mora, Elizabeth A. Bagshaw, Michael R. Prior-Jones, Mauro S. Ferreira Santos, Aaron C. Noell, Peter A. Willis

**Affiliations:** † NASA Jet Propulsion Laboratory, 53411 California Institute of Technology, 4800 Oak Grove Drive, Pasadena, California 91109, United States; ‡ Planetary Science Institute, Tucson, Arizona 85719, United States; § 423125 Royal Canadian Geographical Society, Ottawa, ON K1M 2K1, Canada; ∥ Blue Marble Space Institute of Science, Seattle, Washington 98104, United States; ⊥ School of Geographical Sciences, 1980 University of Bristol, Bristol BS8 1SS, U.K.; # School of Earth and Environmental Sciences, 2112 Cardiff University, Cardiff CF10 3AT, U.K.

**Keywords:** capillary electrophoresis, contactless conductivity detection, in situ chemical analysis, environmental monitoring, instrument automation

## Abstract

In this paper, we report a fully automated, end-to-end (sample-in/data-out) capillary electrophoresis system. The system’s dimensions conform to the cylindrical shape and power/data requirements of the science payload compartment of the Exobiology Extant Life Surveyor (EELS), a snake-like robot capable of autonomously navigating challenging terrain on Earth or other worlds. The capillary electrophoresis system is equipped with three contactless conductivity detectors: two dedicated to analyte detection and one for characterizing bulk sample flow. The system enables simultaneous detection of cations and anions, including K^+^, Na^+^, Ca^2+^, Mg^2+^, Cl^–^, and SO_4_^2–^, at submicromolar concentrations. For the first time, we deployed and demonstrated autonomous capillary electrophoresis operation on a glacier with the system tested in three environments: on-ice, partially submerged in an active stream, and fully submerged in a glacial pond.

## Graphical abstract

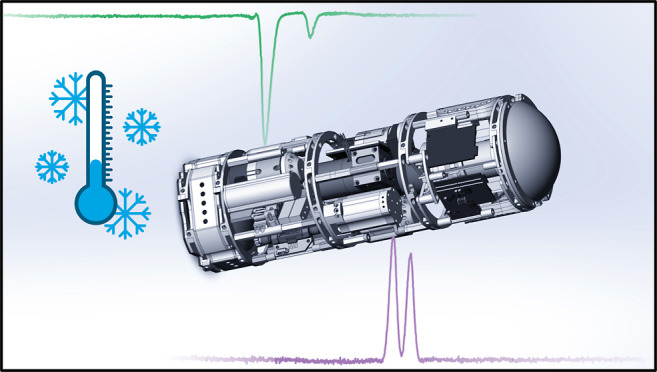

Ocean worlds are defined as planets or moons that contain an existing liquid ocean either on the surface or in the subsurface, and which may or may not be global.^1^ In the Solar System, there are currently several confirmed ocean worlds: Europa, Ganymede, Enceladus, and Titan with evidence for even more.^1,2^ These ocean worlds are often highlighted as compelling destinations for planetary science investigations focused on habitability and the search for life as many appear to satisfy the requirements for a habitable environment for life as we know it: liquid water, chemical building blocks including organic molecules, and energy sources.^3^ Enceladus, a tiny moon of Saturn, is one such ocean world that meets these criteria.^4–6^ At Enceladus, an ice shell ranging from approximately 25 km in thickness at the north pole^7^ to perhaps only a few kilometers thick at the south pole^8^ covers a global liquid water ocean tens of kilometers deep.^9^ Notably, access to ocean materials at Enceladus does not require digging or melting through the ice shell as a plume of ocean-derived particles and vapor actively vents from four large cracks in its south polar terrain.^10–15^

While meaningful science investigations could be performed from measurements collected in orbit or on flybys of Enceladus, addressing astrobiology questions in a robust and comprehensive manner will most likely require a mission with a landed component, enabling larger sample volumes to be collected and analyzed, greatly improving the amounts of putative biosignatures available for detection.^16,17^ For this purpose, the Exobiology Extant Life Surveyor (EELS) concept has been designed—a high-mobility snake-like robot specifically tailored to navigate the ice conduits on Saturn’s moon Enceladus and capable of traversing many other challenging terrains.^18^ The current Earth-based EELS mobility prototype platform is 4 m long and consists of a chain of identically sized modules, each fitted with a propulsive screw designed to move across ice and connected together via novel two-axis bend-twist joints that enable the robot to adopt various 3D shapes and gaits. Throughout the length of the body of future versions of the robot are payload volumes to accommodate scientific instruments. Capable of moving across rough icy terrains on the surface and descending vertical ice conduits while resisting plume forces,^18–21^ the EELS robot is a possible candidate for the future exploration of Enceladus’ ocean.

One of the potential instruments that could be integrated into the EELS robot or a similar platform is a capillary electrophoresis (CE) system. CE is a high efficiency separation technique for molecules in solutions that allows the analysis of a wide range of inorganic and organic compounds. Because CE only requires small amounts of sample (10–100 μL) and liquid reagents (10 s of mL), it is well-suited for in situ analysis. Another advantage of CE is its compatibility with multiple detection systems, which allows tailoring of the instrument to a specific set of analytes. CE allows sensitive analysis of the two most well-recognized biosignatures for life detection: amino acids and fatty acids.^22^ CE coupled to laser-induced fluorescence (LIF) allows analysis of trace amounts of chiral amino acids,^23^ while CE coupled to mass spectrometry allows for the analysis of fatty acids^24^ and a wide range of other ionizable organics essential for life.^23–26^

Another detector particularly relevant for spaceflight missions is capacitively coupled contactless conductivity detection (C^4^D).^27^ C^4^D is advantageous due to its simplicity and ability to detect any charged analytes. We have developed multiple CE-C^4^D methods that are complementary to the ones aimed at biosignaling detection (amino acid and fatty acids). These methods allow the detection of inorganic ions (i.e., sodium, calcium, etc.) as well as inorganic anions such as chloride, phosphate, sulfate, among others. Thus, CE-C^4^D allows us to determine the chemical context of the samples as well as habitability indicators.

More importantly, CE-C^4^D instrumentation can be miniaturized and operated remotely which allows its deployment to investigate extreme environments on Earth, in order to study chemical weathering processes in icy habitats that are otherwise extremely challenging to measure.^28,29^ The capabilities of CE-C^4^D can therefore address various habitability assessment questions through identification and quantitation of potential inorganic habitability indicators, the concentrations and composition of which inform ocean chemistry and transport.^1,30^

We recently demonstrated a fully automated submersible CE-C^4^D system, that was validated by analyzing ocean samples in situ.^31^ In this work, we demonstrate an automated CE-C^4^D system that is ∼1/5 the length of a previous miniaturized CE system,^31^ enabling it to fit into a module of a future EELS robotic platform (Figure 1). The isolated system was successfully operated during a field campaign in a relevant ocean world analogue environment, Athabasca Glacier (Canada) to simultaneously measure cations and anions in glacier meltwater. The details of the instrument development and operation, the results of cation and anion measurements from different glacier surface environments, and the implications for future Earth science and astrobiology investigations implementing CE-C^4^D instrumentation are presented here.

**Figure 1:**
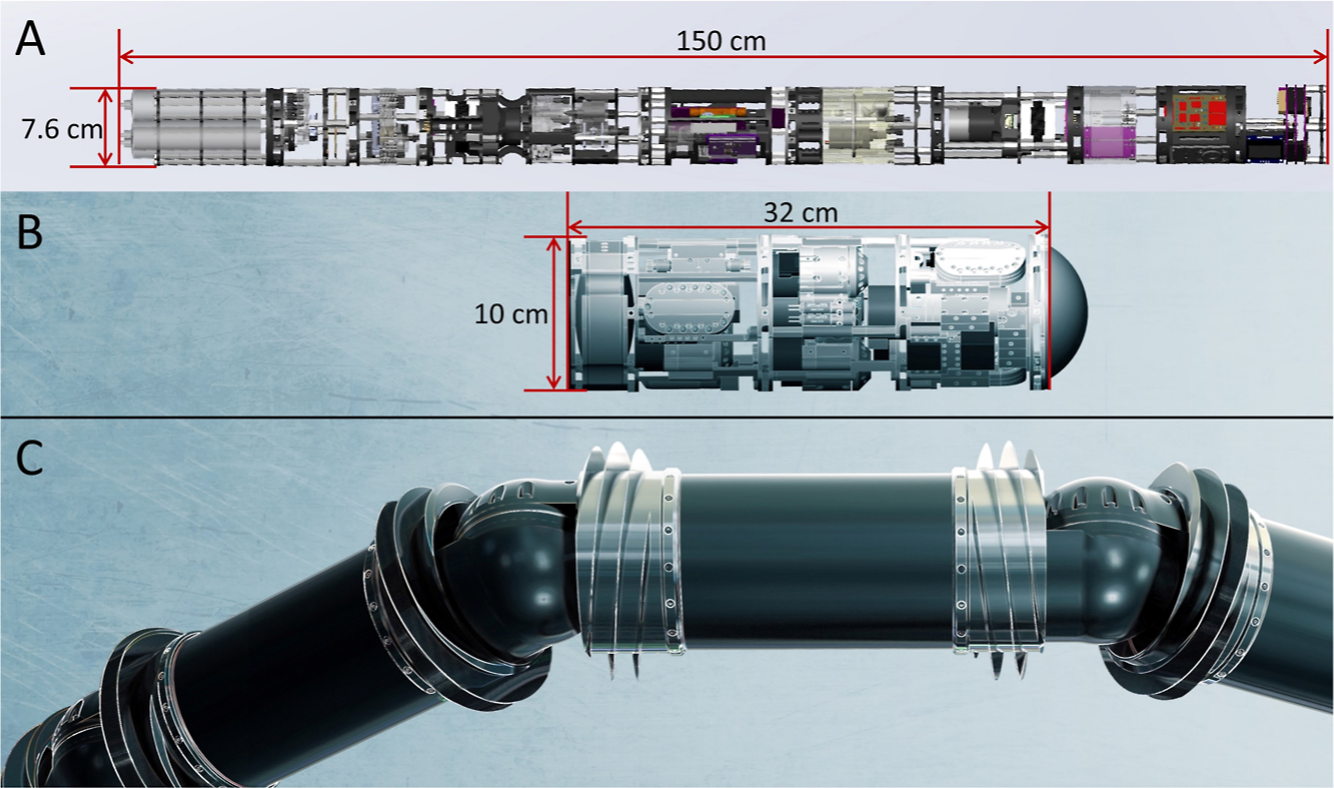
CAD models of the systems developed in this work in reference to previous work. (A) Previous underwater CE system (150 cm long, 7.6 cm in diameter).^31^ (B) Current, miniaturized CE-C^4^D system (32 cm long, 10 cm in diameter). (C) Conceptual model of a proposed module incorporated into the EELS robot designed for field tests at terrestrial analog sites. The model’s scale in panel (A) is approximately two times smaller compared to panels (B and C).

## Results and Discussion

The CE-C^4^D instrument was specifically created to fit within a science payload segment of the EELS robotic snake (Figure 1), which meant reducing the previous instrument volume by a factor of 2.7, from a 6801 mL (150 cm × 7.6 cm diameter) to 2512 mL (32 cm × 10 cm diameter).^31^ Along with a nearly 3-fold reduction in volume, the system also needed to improve its limit-of-detection to submicromolar levels to enable use on low ionic strength glacial melt as opposed to ocean water. The combination of these challenges led to a complete rebuild and redesign of the system with key enabling upgrades described next.

The overall instrument was rearchitected to contain four functional segments, more efficiently combining subsystems to reduce tubing and electrical harnessing lengths and connector volume. The first segment houses reagent and waste storage bags along with the automation electronics. The second and third segments contain the fluidic routing and pneumatic components, respectively. Finally, the fourth segment holds all components required for CE separation, including high voltage power supplies.

The CE segment itself was completely redesigned, with key upgrades to sample injection, HV application, and capillary thermal control. To reduce the volume of reagents needed by half, the sample injection valve was placed in the middle of the separation capillary, allowing both the cationic and anionic profiles to be separated in a single run. This change in sample injection architecture further required a change in high voltage application, switching from a single power supply opposite the injection valve to two high-voltage (HV) power supplies: one positive and one negative at each of the HV reservoirs. With this approach, the entire 14 kV effective voltage could drive the same quality of electrophoretic separation, while the hardware never experienced a voltage difference from ground of greater than 7 kV (positive or negative). This setup allowed the same quality of separations in a more confined space. The reduction in absolute voltage isolation that the system needed to be capable of allowed further size reduction and improvement in capillary temperature regulation by switching to a solid-state approach.^32^ With the reduced possibility of HV breakdown, copper tubing could be used as a heat sink, with the separation capillaries wrapped directly around it in a highly compact coil. This was further aided by the low current of the separation method used to improve the limits-of-detection, discussed below.

Additional improvements to the fluidic routing were also critical to overall size reduction by reducing reagent/waste volumes, as well as the size of the fluidic routing segment. The increase in the reagent usage efficiency and reduction in the volume of both the reagent and waste reservoirs were achieved by switching from latching to rotary valves, eliminating significant dead volume and the need for further operational rinses. This switch was possible due to the use of custom-designed miniaturized 3D-printed rotary valves.^33^ These valves are suitable for low pressure (≤40 psi) pneumatic and fluidic operations providing customizable and selectable port-to-port connections.

The improved detection limits required to analyze glacier water containing small amounts of dissolved salts (submicromolar to tens of micromolar concentrations) were achieved by increasing the injection volume and reducing the conductivity of the background electrolyte.^28,34^ Instead of the 8 nL injection cavity used in the previous system, we used a 30 nL cavity.^31^ In standard applications, CE-C^4^D has a detection limit of approximately 1 μM for monovalent inorganic cations, which serves as the baseline level. To further increase sensitivity and achieve LODs compatible with glacial water analysis, we used a diluted low conductivity background electrolyte consisting of 0.25 mM acetic acid (pH 4.2). This allows for minor changes in conductivity (due to the presence of a low concentration of ions) to be detected. We calibrated the instrument to analyze both cations and anions, specifically K^+^, Na^+^, Ca^2+^, Mg^2+^ for cations, and Cl^–^, SO_4_^2–^ for anions. Calibration information is provided in Table 1.

**Table 1:** Calibration Information

analyte	^a^tested range (μM)	slope (μM^–1^)	*R* ^2^	repeatability (%)	LOD (μM)
K^+^	0.415–333	1.0 × 10^–4^	0.999	2.8	0.830
Ca^2+^	0.415–333	3.8 × 10^–4^	0.998	1.5	0.415
Mg^2+^	0.415–333	3.7 × 10^–4^	0.998	3.3	0.415
Na^+^	0.500–200	2.6 × 10^–4^	0.999	5.1	0.500
Cl^–^	0.415–333	2.9 × 10^–4^	0.999	2.2	0.415
SO_4_^2–^	0.250–100	5.7 × 10^–4^	0.999	1.8	0.250

a The number of calibration points was 7 for cations and 6 for anions.

All ions were detected at the smallest prepared concentration, except K^+^, which was detected at 0.830 μM. All analyses were done in triplicates. Separate calibration curves are represented in Figures S1 and S2. To compensate for peak migration time shifts, we used an electroosmotic flow marker, which was visible as a dip in the electropherogram containing the cation peaks (illustration of anions, cations, and electroosmotic flow is depicted in Figure S3). Limits of quantification were 2.5, 1.3, 1.3, 1.5, 1.3, and 0.8 μM for K^+^, Ca^2+^, Mg^2+^, Na^+^, Cl^–^, and SO_4_^2–^, respectively.

### Instrument Deployment

We performed three levels of testing: (1) placing the CE-C^4^D instrument on the ice, sampling from Falcon tubing filled with nearby sample collected by human operators, and conducting the analysis (Figure 2A); (2) deploying the CE-C^4^D instrument in an active channel with flowing water, autonomously sampling directly from the stream, and performing the analysis (Figure 2B); and (3) fully submerging the CE-C^4^D instrument in a > 100 cm deep glacial meltwater pond, followed by automated sampling and analysis (Figure 2C).

**Figure 2:**
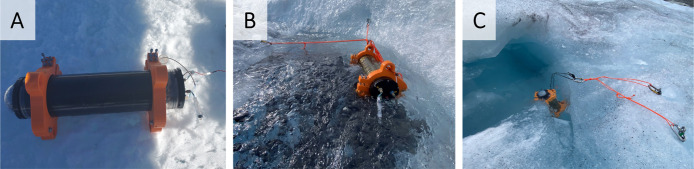
Testing of the CE-C4D instrument on a glacier. (A) Deployment on ice (GS1). (B) Partial submersion in a stream (MSS4). (C) Full submersion in the glacier pond (P11) (sampling map is given in Figure S6).

During the first level of testing, we confirmed that the instrument could operate in a portable manner, similar to a laboratory environment, albeit with some differences such as the colder and fluctuating temperatures and the uneven surface of glacier ice compared to a flat laboratory table.

In the second level of testing, we confirmed that the instrument was waterproof and could operate without physical assistance from an operator, sampling water directly from the stream. Due to the orientation within the active channel, it was more convenient to deploy the instrument with one HV reservoir positioned higher than the other. Slight siphoning effects were observed, which have been observed and characterized in HV electrode reservoirs.^35^

At the third level of testing, the deployed instrument was fully submerged, confirming that it was watertight. Moreover, the instrument was placed in a nearly vertical orientation, where the CE module was facing upward. The CE separations ran without any issues, confirming that the system can operate in multiple orientations. Also, while performing the last repetition, we had to retrieve the instrument due to timing constraints performing field operations and carry it over the glacier by hand and then place it in a moving vehicle while the analytical run was still occurring. Despite these perturbations, the CE data were not altered, confirming that the instrument can operate while in motion (similar to the submersible CE-C^4^D predecessor instrument which operated successfully while subjected to wave motion in the ocean). Hence, a robotic vehicle will only have to stop for sampling (if this requires stopping) and then can continue its traverse while the CE-C^4^D analysis is in progress.

Another important observation is that when the CE-C^4^D system was in direct contact with glacial water, the instrument consumed more power to regulate the temperature. This depleted the battery twice as fast. The full analytical cycle (priming, sampling, conditioning, three 50 min long analyses, and shutdown) consumed 32 Wh of energy when fully submerged in a glacial pond. Full battery energy capacity is ∼38 Wh. Future work will optimize the overall thermal design of the instrument to reduce energy needs for use in the intended environments.

### Experiments and Data

Three samples were analyzed by the CE-C^4^D instrument in meltwater ponds or marginal streams (Table 2), while two additional samples were collected from the glacier but only analyzed in the lab due to time constraints. The first two samples (GS1 and the pond at P11) represent supraglacial water, whereas the fourth sample (MSS4) represents surface water runoff from the nearby mountain. A detailed sampling map is included in the Supporting Information (Figure S6).

**Table 2:** Sampling Details^a^

test level	dilution	name	date	time	coordinates
1	1	GS1	9/21/2023	9:30	52.19799, −117.24629
2	10	MSS4	9/26/2023	10:00	52.19926, −117.24646
3	1	P11	9/25/2023	9:30	52.19850, −117.24309
^b^	10	MSS7	9/26/2023	14:40	52.19972, −117.24638
^b^	1	M8	9/27/2023	9:20	52.19812, −117.24518

a Abbreviations: GS, glacier stream; MSS, marginal supraglacial stream; P, pond; M, moulin.

b CE analysis performed in the laboratory with the CE-C^4^D instrument.

To ensure correct sampling, the bulk sample flow was recorded by using a C^4^D detector positioned on the sample line. The C^4^D detector measures electrical impedance of the liquid sample at a 32 kHz square wave that can be recalibrated and expressed as a selected dissolved salt concentration. Baseline changes that indicated different solutions reaching the detection window of the C^4^D detector on the sample line were monitored. In all cases, a baseline change was observed during sampling, confirming that the sample had entered the sampling line. The instrument stabilized after <5 min and recorded consistent values (unitless) for the different sample types: GS1, −0.229; P11, −0.217; MSS4, −0.155; MSS7, −0.153; M8, −0.210. In the future, this information will be used to predict bulk ionic concentration and facilitate autonomous sample dilution, allowing for optimal CE-C^4^D data collection with small concentrations being detectable and large concentrations not saturating the detector.

Figure 3 shows the electropherograms of the various samples. Across all analyzed samples, four distinct peaks were observed in the cationic electropherograms, identified as different cations: K^+^, Ca^2+^, Mg^2+^, and Na^+^. The Na^+^ peak was not baseline separated from Mg^2+^ and is seen as a shoulder, yet peak fitting and integration could be performed (standard separation is shown in Figure S4). In the anionic electropherograms (Figure 3B), only two anions were identified: Cl^–^ and SO_4_^2–^. Separation of the anion mixture is depicted in Figure S5.

**Figure 3:**
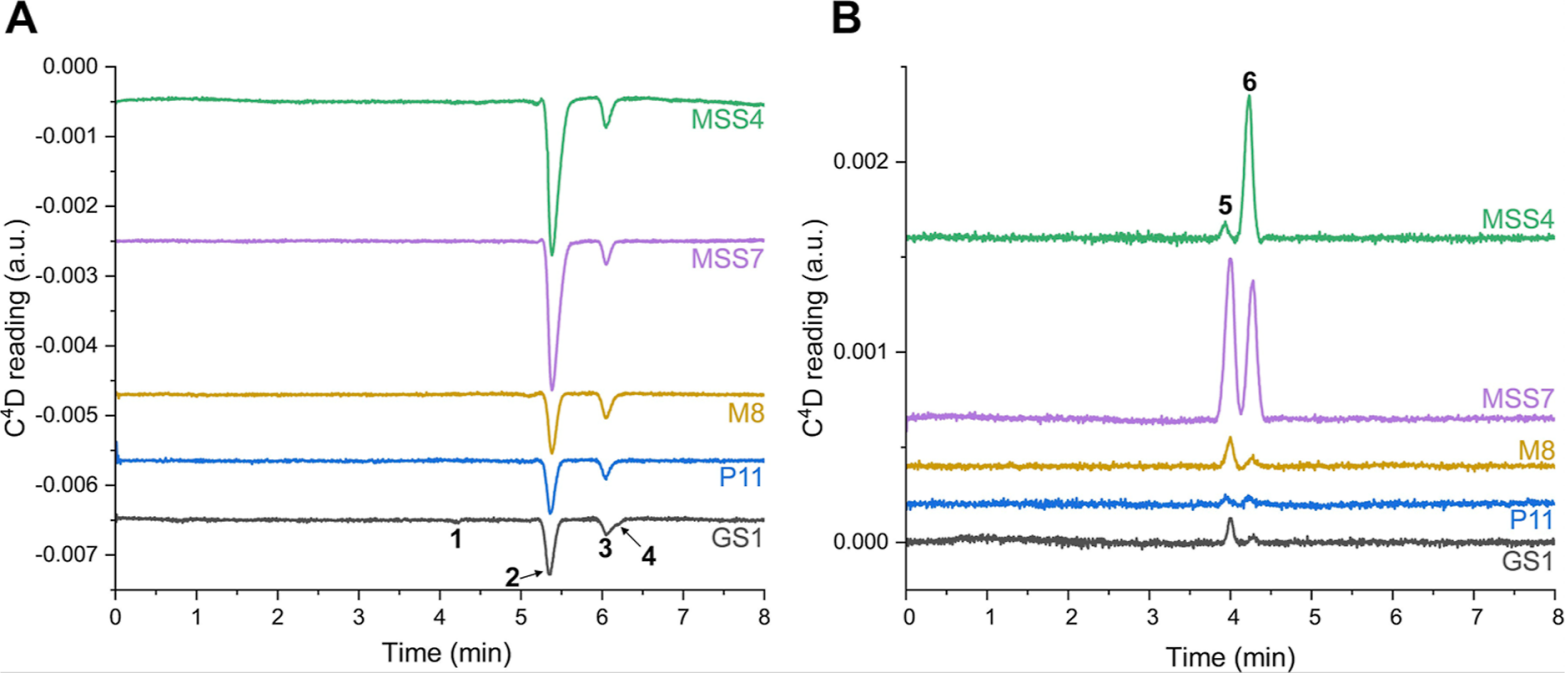
CE-C^4^D electropherograms of different samples collected and analyzed autonomously during the field campaign at Athabasca Glacier in Canada. Cations—(A) and anions—(B). Peaks identified as 1—K^+^, 2—Ca^2+^, 3—Mg^2+^, 4—Na^+^, 5—Cl^–^, 6—SO_4_^2–^. Conditions: BGE—0.25 mM acetic acid, *L*_tot_—40 cm, *L*_eff_—32 cm, temperature −10 °C, voltage for cations −7.2 kV, voltage for anions +7.2 kV, C4D detection at 3.3 V, square wave at 32 kHz, electropherograms autozeroed upon start of analysis. Peak migration time corrected. Baselines of each electropherograms are offset for better viewing. Abbreviations: GS, glacier stream; M, moulin.

**Table 3:** Concentration (μM) of Cations and Anions in Different Samples and Literature

location-Glacier	sample/reference	sample type	ion concentration (μM)	K^+^	Ca^2+^	Mg^2+^	Na^+^	Cl^–^	SO_4_^2–^
Canada. Athabasca	MSS4	I	704.5	N/D	508.0 ± 7.6	77.9 ± 2.6	N/D	1.8 ± 0.1	9.4 ± 0.2
Canada. Athabasca	MSS7	I	717.4	3.2 ± 0.1	492.4 ± 7.4	55.5 ± 1.8	N/D	23.9 ± 0.5	9.4 ± 0.2
Canada. Athabasca	M8	II	4.9	N/D	16.5 ± 0.3	7.6 ± 0.3	N/D	3.2 ± 0.1	0.5 ± 0.0
Canada. Athabasca	P11	II	6.6	N/D	13.5 ± 0.2	4.8 ± 0.2	N/D	N/D	0.4 ± 0.0
Canada. Athabasca	GS1	III	9.7	1.8 ± 0.1	14.1 ± 0.2	4.6 ± 0.2	1.8 ± 0.1	2.1 ± 0.0	0.3 ± 0.0
Svalbard. Aldegondabreen, Gronfjordbreen	ref 36	IV	N/A	5.6	5	7.4	27	N/A	N/A
Norway. Jostedalsbreen, Bodalsbreen	ref 36	IV	N/A	18	19	40	9.6	N/A	N/A
Nepal. Khumbu, Ngozumpa	ref 36	IV	N/A	19	25	8.2	8.7	N/A	N/A
Greenland. Leverett	ref 37	III	N/A	0.7	N/A	2	3	2.9	0.4
Antarctica. Canada	ref 37	III	N/A	3.6	47	4.6	15	19	4.7

I—Marginal supraglacial stream; II—Supraglacial pond; III—Supraglacial stream; IV—Supraglacial ice. N/A—Not available. N/D—Not detected.

Table 3 presents the determined cationic and anionic compositions of the samples. The predicted ionic strength, measured by using the bulk sample flow with C^4^D, aligns with the findings of the CE-C^4^D analysis of the individual species.

From the cationic profile, it is possible to distinguish two classes of samples: those with a low concentration and those with a higher concentration. The low concentration samples are the supraglacial waters—GS1, P11, and M8, with Ca^2+^ and Mg^2+^ in the low μM range (∼5 to 16 μM). The higher concentration samples are the surface runoff water from the fourth, and MSS7s showed higher concentrations of both Ca^2+^ and Mg^2+^ ranging from ∼55 to 508 μM. This is likely due to the proximity of the supraglacial streams to the margin of the ice sheet (Figure S6) as the local geology includes exposures of limestone and shale, which would be consistent with these elevated ionic values in runoff.^38^

From the anionic profile, the samples can be classified similarly: low concentration and higher concentration. The levels of submicromolar concentration of Cl^–^ and SO_4_^2–^ were detected in the low concentration samples. Low μM ranges were detected in both higher concentration samples (MSS4 and MSS7, consistent with the higher cationic loads).

The two classes of samples are indicative of the degree of chemical weathering that has occurred prior to sampling; the supraglacial samples have had little interaction with any source of solute so are close to pure ice melt, whereas the “Marginal supraglacial stream” samples are more influenced by the local geology as there is significant debris cover along the margin of the glacier (Figure S6) from which meltwater is being sourced, and solute is acquired through mineral weathering. The concentrations of the supraglacial sample ions measured are comparable to other glacier surface environments sampled worldwide (Table 3), which have a similar ionic profile.^36,37^ The comparative data confirm that the instrument performs at least as well as the existing manual sampling methods (ICP–MS or ion chromatography), without requiring sample removal, filtering, refrigerated transport, and a significant delay before concentration data can be obtained. This enables real- or near-real-time decision-making in the field, indicating subsequent sampling locations.

The field tests demonstrate that the instrument can differentiate between the chemical characteristics of different supraglacial environments, even in extremely low ionic concentration samples. They also show that in situ measurement of the chemical composition of meltwater is possible, demonstrating the potential for sampling inaccessible icy environments on Earth and beyond.

## Conclusions

For the first time, an automated capillary electrophoresis system with a capacitively coupled contactless conductivity detector was deployed and operated on a glacier in three different environments: (i) on ice, (ii) partially submerged in an active channel, and (iii) fully submerged in a pond on the glacier. The instrument was able to collect samples and complete analysis for cations and anions while being fully submerged, all without user intervention. This is the first demonstration of its kind. The system was designed to meet the form factor and power/data requirements of an Exobiology Extant Life Surveyor’s payload segment, making this demonstration the first step toward implementing this technology in such a mission.

## Materials and Methods

### Analytical Instrumentation

The CE-C^4^D instrument is a cylinder-shaped device that has a length of 32 cm and a diameter of 10 cm. A schematic diagram of the CE-C^4^D instrument’s submodules is given in Figure S7. It has two C^4^D detectors for detecting anions and cations, and it has a third C^4^D detector for measuring bulk sample flow. The instrument has two high voltage power supplies (Q101-5 and Q101N-5, XP Power, United states) capable of supplying voltage up to 20 kV. Two heating pads (14 cm × 5 cm) dissipated 11 W, ensuring that the temperature within the instrument was regulated and stable. The instrument’s fluidic and pneumatic architecture is similar to a previously reported study.^31,39^ Sample injection is done with an injection valve that has a cavity of 30 nL.^31,39^ Background electrolyte (BGE) and water are drawn from the reagent bags and delivered to the CE module via a piston pump. High voltage decoupling is done pneumatically as reported in previous work.^31,35^ For the deployment, the instrument is supplied with a 4S1P Li-ion battery pack that can provide enough power for a full working day of operations. For tests that did not require complete sealing of the instrument (deployment on ice), an external 4S3P Li-ion battery pack was used.

In this configuration, the instrument housed 25 mL of BGE and 25 mL of water that could be expelled into a 50 mL waste storage bag after use. These volumes allow performing triplicate analysis of at least 8 different samples.

### Chemicals

Required stock solutions were prepared on the 21st of August 2023 and shipped to Athabasca Glacier for use. Only analytical grade reagents were used in this work. Acetic acid was purchased from Thermo Fisher Scientific Inc. (Waltham, MA). Potassium chloride, calcium chloride, magnesium chloride, and sodium sulfate were obtained from Sigma-Aldrich (St. Louis, MO). Solutions were prepared in lab with water purified by a Milli-Q system (USA) producing deionized water (>18 MΩ·cm).

### Storage

Chemicals and instrumentation were delivered within plastic bulk containers on a trailer from Pasadena, USA (CA) to the parking lot close to Columbia Icefield Glacier Discovery Center, Alberta, Canada on 10th of September. On 12th of September, plastic containers were delivered to the glacier by helicopter and left on ice until 27th of September. Instrumentation was kept in a Pelican case, and chemicals were kept inside secondary polypropylene containers. Every morning, chemicals and instrumentation were taken from the plastic containers and in the evening, chemicals and instrumentation were put back in the plastic containers which prevented solutions from freezing.

### Procedures

Once initiated, the instrument is fully automated and performs an end-to-end analytical process without the input of a human operator.

The analytical cycle was subdivided into 3 smaller protocols: (a) sampling, (b) system rinsing, and (c) separation. Protocols were pre-loaded into the microcontroller’s memory. All of the protocols had a power-reset and initialization function that was run before actual valve and pump actuations.

#### Sampling

Samples are infused into the instrument via a Whatman Puradisc 25 poly­(ether sulfone) membrane 1 μm pore size filter (Cytiva, UK). On startup, the sample delivery path is cleaned with water, where the whole process uses 1200 μL. Then, water is removed by purging the lines with air at 10 psi for 15 s. 750 μL of the sample is used to rinse the respective lines; the sample is then directed to the waste bag. If dilution is needed, the instrument mixes the sample with water at a ratio 1:9 and delivers it to the injection valve.^31^ After the prepared sample (aliquot) is delivered to the injection valve, other fluidic lines are cleared of liquid by purging with air at 10 psi for 15 s.

#### System Rinsing

The main line, which is the line that shares the sample and the BGE path, is cleaned with a 600 μL portion of water. Then, 2800 μL of BGE is used to clean lines connecting HV reservoirs and fill them. Next, the separation capillary together with sample injection cavity is rinsed for 17 min. After the cells were rinsed, the HV decoupling procedure is performed by purging the lines with air.

#### Separation

The sample injection cavity is rinsed with the prepared sample for 8 s at 6 psi. Then, the injection valve is rotated to the separation position, bringing 30 nL of the sample in contact with the BGE within the separation capillary. High voltage is applied, and the detector starts recording the signal.

#### CE Parameters

The total capillary length (*L*_tot_) is 40 cm, while the effective capillary length (*L*_eff_) is 32 cm. The inner diameter of the capillary is 50 μm, and the outer diameter is 365 μm. Applied voltage per capillary is 7.2 kV (for cations, negative; for anions—positive). Sample injection volume is 30 nL. Acetic acid (0.25 mM) was used as the BGE. Before sampling, the capillary was rinsed with BGE for 17 min, and no capillary rinses were done between repetitions. The analysis time was set to 50 min to make sure that electroosmotic flow flushes out all the sample to avoid cross-contamination.

### Instrument Control

To control the instrument, a purpose-built remote control was designed and used. To make sure that we have radio frequency penetration through several meters of ice and water, we decided to use 915 MHz LORA. The remote control has numbers 0–9, a button for deleting, and a button for sending a command. All commands are integer numbers. Three-digit integers (1–304) were reserved as subroutines. The subroutines mimic actual steps that a chemist would perform in the laboratory, for example, “purge main line”, “dilute sample at ratio 1:10”, or “perform HV reservoir inlet HV decoupling”. 4-Digit integers (1100–8300) were reserved for simple functions such as “switch on pneumatic pump”, “rotate injection valve to separation position”, etc. 5-digit commands were reserved for loading, starting, and stopping protocols (10,000–19,999). Protocols are collections of different subroutines. For starting and stopping compound protocols, we used this range of integers: 20,000–29,999. Compound protocols are collections of multiple protocols.

This strategy allows control of the instrument and reading of its status after deployment without using a computer. All protocols were loaded from, and analytical data collected to, an SD card that resided within the CE-C^4^D instrument electronics.

Before analysis, the priming protocol was run, which takes roughly 1200 s to complete. The protocol ensures that all delivered liquids, including samples, are metered. Depending on the difference between the ambient temperature and the inside of the instrument, there might be variations in the flow rates. However, fluid metering takes the volume into account, which results in consistent volumes through the pump.

## Supplementary Material



## Notes

Calibration curves and sampling map (PDF)

## Abbreviations

CE: capillary electrophoresis
C^4^D: capacitively coupled contactless conductivity detection
EELS: exobiology extant life surveyor
HV: high voltage.
