# Supplementary material for: In Situ Analytical Chemistry Laboratory for the Exobiology Extant Life Surveyor
Source: ACS Meas Sci Au. 2026 Mar 12;6(2):421–9. doi: 10.1021/acsmeasuresciau.5c00173 (PMC13087958; doi:10.1021/acsmeasuresciau.5c00173)
Supplement: Supplementary file 1 [file tg5c00173_si_001.pdf]

© 2025. All rights reserved.

Supporting information

In Situ Analytical Chemistry Laboratory for the Exobiology Extant Life Surveyor

Tomas Drevinskas[1], Morgan L. Cable[1], Christian Stenner[2], Alex S. Gardner[1], Michael Paton[1], Michael J. Malaska[1], Sarah Cruz[1], Richard Rieber[1], Rachel Etheredge[1], Matthew Robinson[1], Masahiro Ono[1], Maria F. Mora[1], Elizabeth A. Bagshaw[3], Michael R. Prior-Jones[4], Mauro S. Ferreira Santos[1], Aaron C. Noell[1], Peter A. Willis[1]

[1]NASA Jet Propulsion Laboratory, California Institute of Technology, 4800 Oak Grove Drive, Pasadena, California 91109, United States

[2]Royal Canadian Geographical Society, Ottawa, Ontario, Canada

[3]School of Geographical Sciences, University of Bristol, UK

[4]School of Earth and Environmental Sciences, Cardiff University, Cardiff, UK

KEYWORDS: Capillary electrophoresis, Contactless conductivity detection

Corresponding Author:
NASA Jet Propulsion Laboratory, California
Institute of Technology, Pasadena, California 91109, United
Email: peter.a.willis@jpl.nasa.gov

© 2025. All rights reserved.

Contents

Figure S1. Cation calibration curves.

Figure S2. Anion calibration curves.

Figure S3. Sampling map in the Athabasca glacier.

© 2025. All rights reserved.

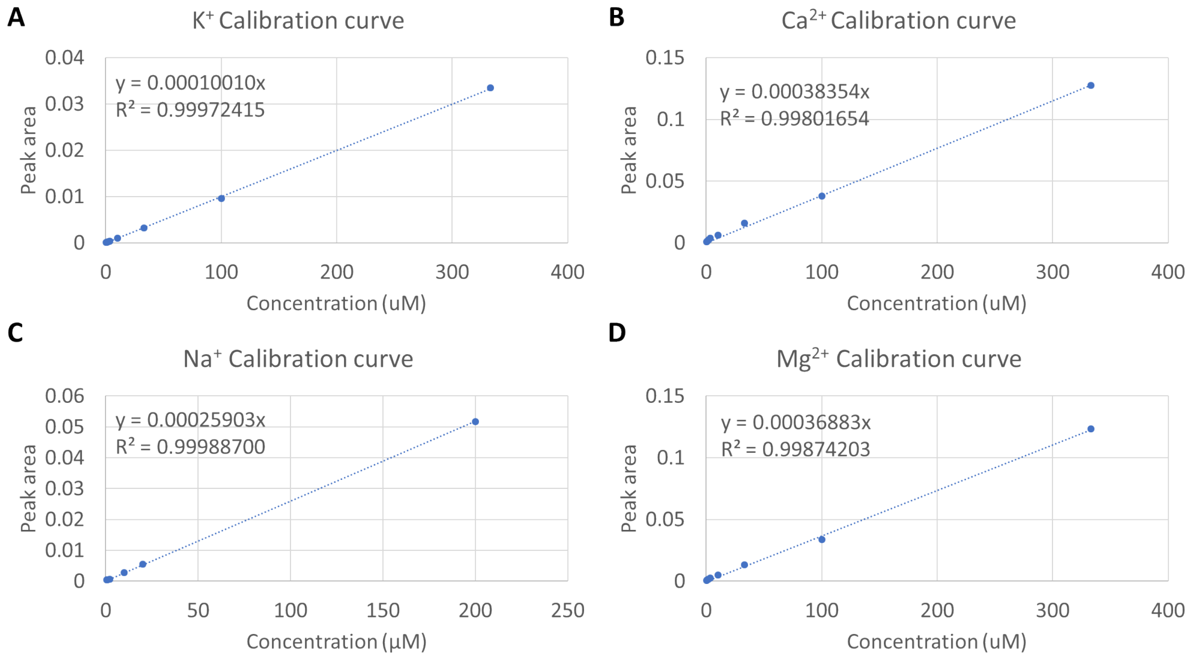

Fig. S1. Cation calibration curves. A) K^+ calibration curve. B) Ca^{2+} calibration curve. C) Na^+ calibration curve. D) Mg^{2+} calibration curve.

© 2025. All rights reserved.

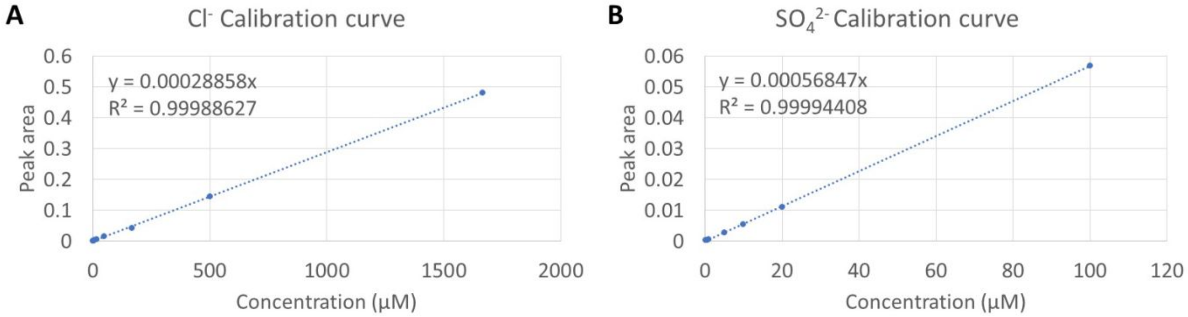

Fig. S2. Anion calibration curves. A) Cl^- calibration curve. B) SO_4^{2-} calibration curve.

© 2025. All rights reserved.

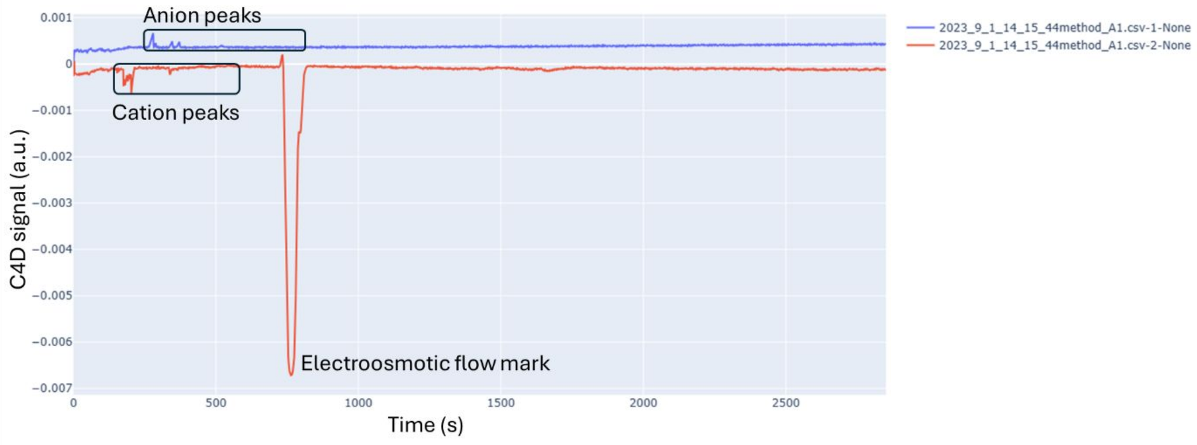

Fig. S3. Depiction of cation peaks, anion peaks and electroosmotic flow in a single run with the developed CE-C^{4}D system.

© 2025. All rights reserved.

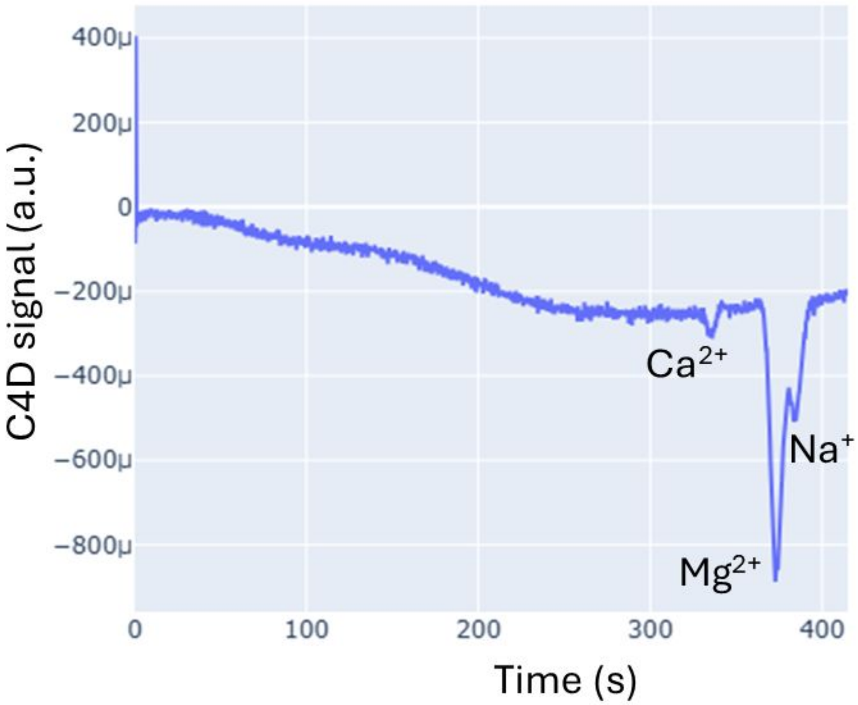

Fig. S4. Raw electropherogram depicting Ca^{2+}, Mg^{2+} and Na^{+} separation.

© 2025. All rights reserved.

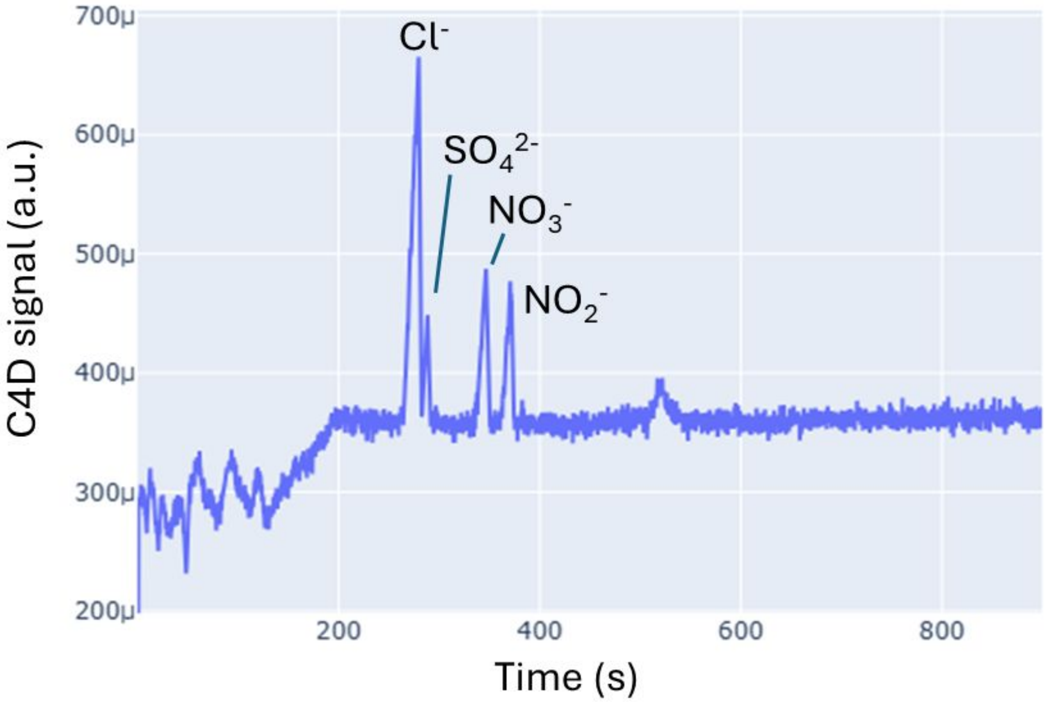

Fig. S5. Raw electropherogram of anion mixture performed with developed CE-C[4]D instrument.

© 2025. All rights reserved.

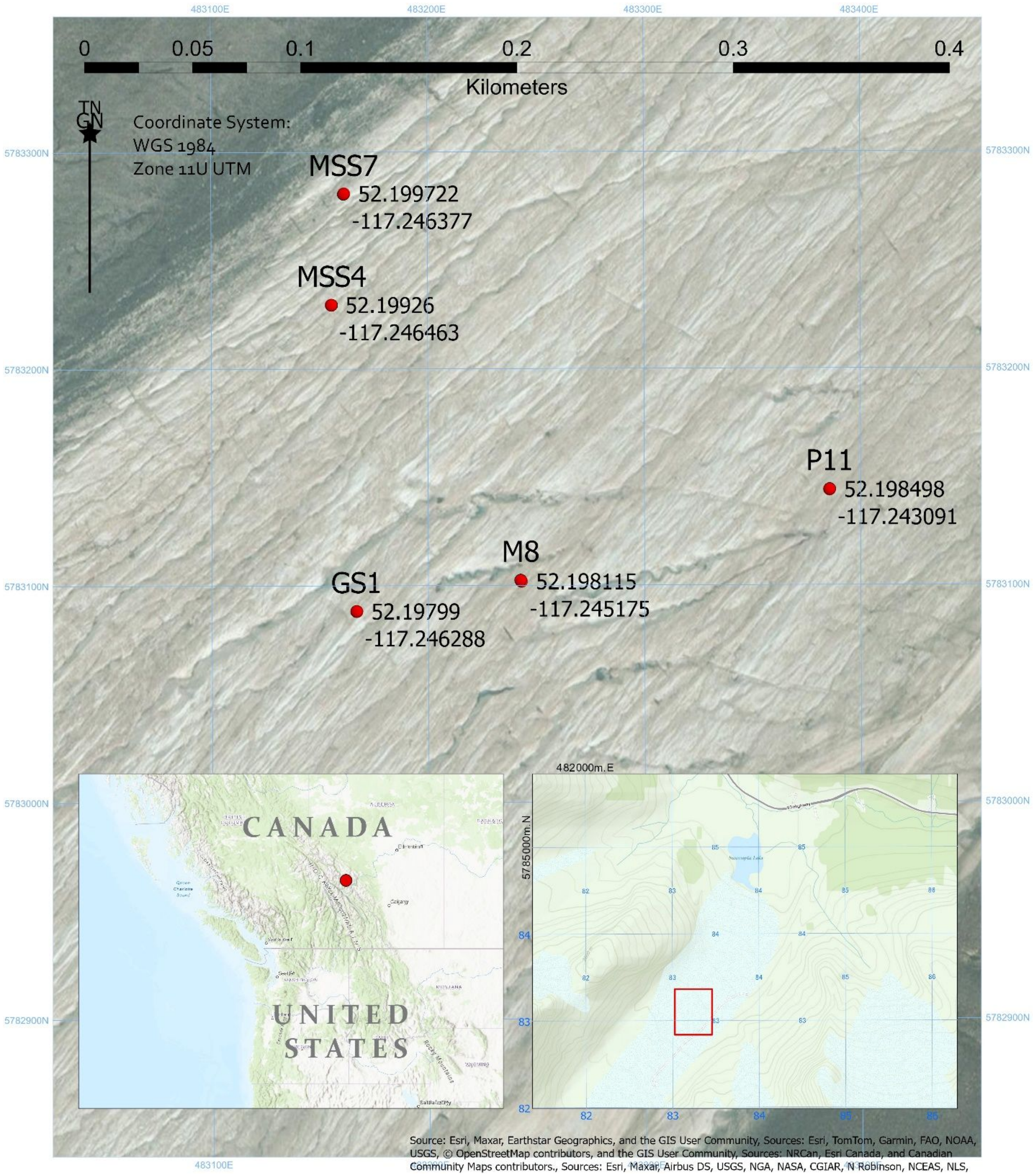

Fig. S6. Sampling map on the Athabasca glacier.

© 2025. All rights reserved.

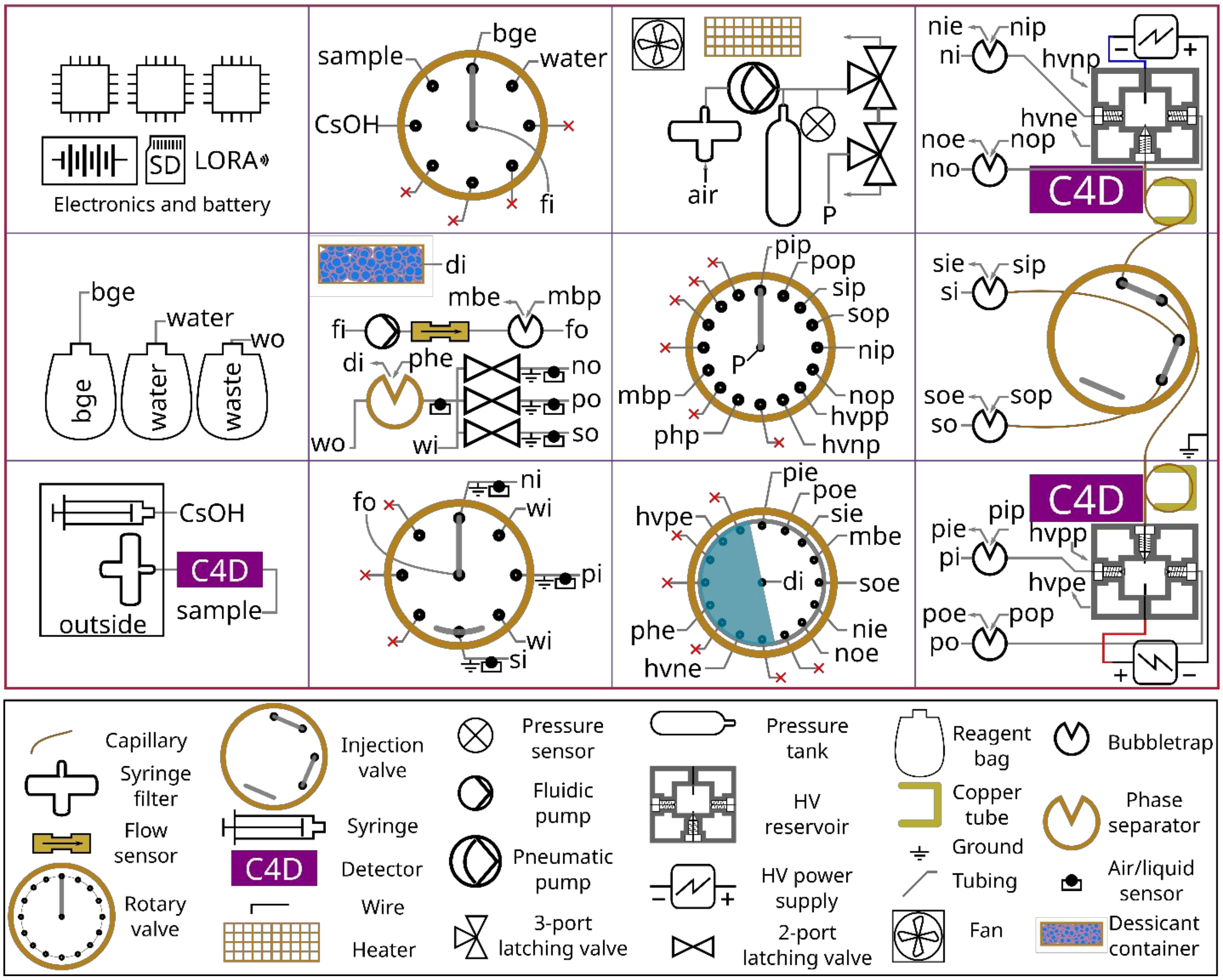

Fig. S7. Schematic diagram (global label style) of CE-C^{4}D instrument's submodules. Annotations: bge – background electrolyte; C4D – contactless conductivity detector; CsOH – Cesium hydroxide; di – dessicant inlet; fi – fluidic inlet; fo – fluidic outlet; HV – high voltage; hvne – high voltage negative (bubbletrap) exit; hvnp – high voltage negative pressure; hvpe – high voltage positive (bubletrap) exit; hvpp – high voltage positive pressure; LORA – long range communication; mbe – main bubbletrap exit; mbp – main bubbletrap pressure port; ni – negative (side) inlet; nie – negative inlet (bubbletrap) exit; nip – negative inlet pressure; no – negative (side) outlet; noe – negative outlet (bubbletrap) exit; nop – negative outlet pressure; P – pressure; phe – phase separator exit; pi – positive (side) inlet; pie – positive inlet (bubbletrap) exit; pip – positive inlet pressure; po – positive (side) outlet; poe – positive inlet (bubbletrap) exit; pop – positive outlet pressure; SD – sd card; si – sample (side) inlet; sie – sample inlet (bubbletrap) pressure; sip – sample inlet pressure; so – sample (side) outlet; soe – sample outlet (bubbletrap) exit; sop – sample outlet pressure; wi – waste inlet; wo – waste outlet.
